# The impact of implant material and patient age on the long-term outcome of secondary cranioplasty following decompressive craniectomy for severe traumatic brain injury

**DOI:** 10.1007/s00701-020-04243-7

**Published:** 2020-02-05

**Authors:** Martina Hamböck, Arthur Hosmann, Rudolf Seemann, Harald Wolf, Florian Schachinger, Stefan Hajdu, Harald Widhalm

**Affiliations:** 1 Department of Biomedical Imaging and Image-Guided Therapy, Division of Nuclear Medicine, Medical University of Vienna, Vienna, Austria; 2 Department of Neurosurgery, Medical University of Vienna, Vienna, Austria; 3 Department of Cranio-Maxillofacial and Oral Surgery, Medical University of Vienna, Vienna, Austria; 4 Department of Orthopedics and Traumatology, Clinical Division of Traumatology, Medical University of Vienna, Waehringer Guertel 18-20, 1090 Vienna, Austria

**Keywords:** Decompressive craniectomy, Implant material, Long-term outcome, Secondary cranioplasty, Traumatic brain injury

## Abstract

**Background:**

Secondary cranioplasty (CP) is considered to support the neurological recovery of patients after decompressive craniectomy (DC), but the treatment success might be limited by complications associated to confounders, which are not yet fully characterized. The aim of this study was to identify the most relevant factors based on the necessity to perform revision surgeries.

**Methods:**

Data from 156 patients who received secondary CP following DC for severe traumatic brain injury (TBI) between 1984 and 2015 have been retrospectively analyzed and arranged into cohorts according to the occurrence of complications requiring surgical intervention.

**Results:**

Cox regression analysis revealed a lower revision rate in patients with polymethylmethacrylate (PMMA) implants than in patients with autologous calvarial bone (ACB) implants (HR 0.2, 95% CI 0.1 to 1.0, *p* = 0.04). A similar effect could be observed in the population of patients aged between 18 and 65 years, who had a lower risk to suffer complications requiring surgical treatment than individuals aged under 18 or over 65 years (HR 0.4, 95% CI 0.2 to 0.9, *p* = 0.02). Revision rates were not influenced by the gender (*p* = 0.88), timing of the CP (*p* = 0.53), the severity of the TBI (*p* = 0.86), or the size of the cranial defect (*p* = 0.16).

**Conclusions:**

In this study, the implant material and patient age were identified as the most relevant parameters independently predicting the long-term outcome of secondary CP. The use of PMMA was associated with lower revision rates than ACB and might provide a therapeutic benefit for selected patients with traumatic cranial defects.

**Electronic supplementary material:**

The online version of this article (10.1007/s00701-020-04243-7) contains supplementary material, which is available to authorized users.

## Introduction

Decompressive craniectomy (DC) is a potentially life-saving procedure performed with the objective of relieving critically raised intracranial pressure (ICP) [1]. Commonly, the necessity of opening the skull arises from a malignant brain swelling following severe traumatic brain injury (TBI). The use of DC remains controversial as it is associated with worse neurological outcomes than a conservative ICP management if performed routinely but might provide a therapeutic benefit for individually selected patients [2, 3].

Once the brain swelling has subsided, the surgical skull defect must be bridged [4]. In the past, the use of autologous calvarial bone (ACB) was considered the gold standard for secondary cranioplasty (CP). However, the treatment success might be limited by high rates of bone flap resorption, varying between 2 and 17% in adults and up to 50% in children [5, 6]. Therefore, alloplastic implant materials including polymethylmethacrylate (PMMA) have become a popular alternative for secondary CP. Interestingly, overall complication rates for PMMA implants range up to 24% and are equivalent to those reported for ACB [7]. In addition to implant-specific properties, there are also other parameters assumed to influence the treatment success of secondary CP including the age, gender, severity of the TBI, timing of the CP, bone flap fragmentation, size of the skull defect, and the occurrence of infections [8–10].

In contrast to DC—which is an emergency procedure—secondary CP aims to improve the patient’s neurological condition and accelerate the physical and social rehabilitation. Therefore, secondary CP needs to be performed in the safest way possible with minimal risk of severe complications. However, current data addressing the safety of secondary CP and the impact of important confounders on the outcome are rather divergent [11]. Due to the lack of standardization, secondary CP is often performed empirically or according to institutional and personal preferences.

This study was designed to assess the impact of patient- and treatment-specific parameters on the long-term outcome of secondary CP as determined by a clinically relevant endpoint: the necessity of revision surgeries due to complications associated with the procedure or the implanted graft. We hypothesized that the treatment success of secondary CP might be influenced by the implant material, gender, patient age, reconstruction interval, severity of TBI, size of the cranial defect, or by a constellation of the aforementioned factors. The identification of significant correlations could help to formulate treatment recommendations in order to minimize postoperative complication rates and improve the rehabilitation of future patients undergoing secondary CP.

## Methods

### Study design

The present study was designed as a retrospective single-center cohort study based on clinical data of patients who underwent secondary CP following DC for severe TBI between 1984 and 2015. Patients were arranged into cohorts according to the occurrence of complications, which required a surgical revision procedure. The conduction of this study was approved by the local ethics committee (ID number: 999/2010).

### Patients

Patients were eligible for this study if they had suffered severe TBI and received DC for the treatment of elevated ICP. Secondary CP had to be performed using either a frozen ACB or a PMMA implant. Patients were excluded if another material has been used for cranial reconstruction or in case of incomplete data.

### Surgical techniques

The preferentially used implant material for secondary CP was ACB. PMMA implants were used in patients with limited availability of the ACB—caused by contamination or fragmentation—or in patients whose DC was performed in another hospital. There were no significant changes in the preservation methods of ACB implants, the surgical techniques, or the materials used for secondary CP over the observed treatment period. The timing of secondary CP was chosen individually according to the patient’s general health condition.

The ACB removed during DC was cleaned from adhering blood and soft tissue using saline solution. It was then sealed in three sterilized plastic bags and preserved in a special deep-freezing system at a mean temperature of − 80 °C. Prior to secondary CP, the frozen autograft was left at room temperature to thaw. After reopening the wound, the defrosted ACB was adapted to fit the skull defect. Following the placement of dural tenting sutures, the autograft was secured in its original position using either small titanium plates and screws or the Craniofix® system and sutures.

For PMMA implants, a malleable titanium mesh was used as a template to form a mold of the patient’s original skull bone. Then, PMMA was mixed and casted into the mold. After hardening, the implant was adapted to create an exact duplicate of the patient’s natural skull contour. The rigid fixation of the PMMA implant was achieved either with small titanium plates and screws or with the Craniofix® system and sutures. Dural tenting sutures were placed routinely.

All patients were operated under general anesthesia. A prophylactic dose of cefuroxime 1500 mg was administered 30 min prior to surgery and continued for 5 days postoperatively. In case of penicillin allergy, patients received clindamycin 600 mg. The patients received a suction drain according to the preferences of the surgeon. The wound was closed layer by layer using a running subcutaneous suture and interrupted skin sutures. Depending on the clinical performance, patients were transferred either to the intensive care unit or to the ward postoperatively.

CCT scans were performed routinely 1 day after the surgery and before discharge. The suction drain was removed within the first 3 days, dependent on the amount of subgaleal fluid. The sutures were removed after 10 days. The first clinical control was scheduled 4 to 6 weeks after CP. Subsequent follow-up examinations and/or CCT scans were arranged individually.

### Variables and outcome measurements

**Fig. 1:**
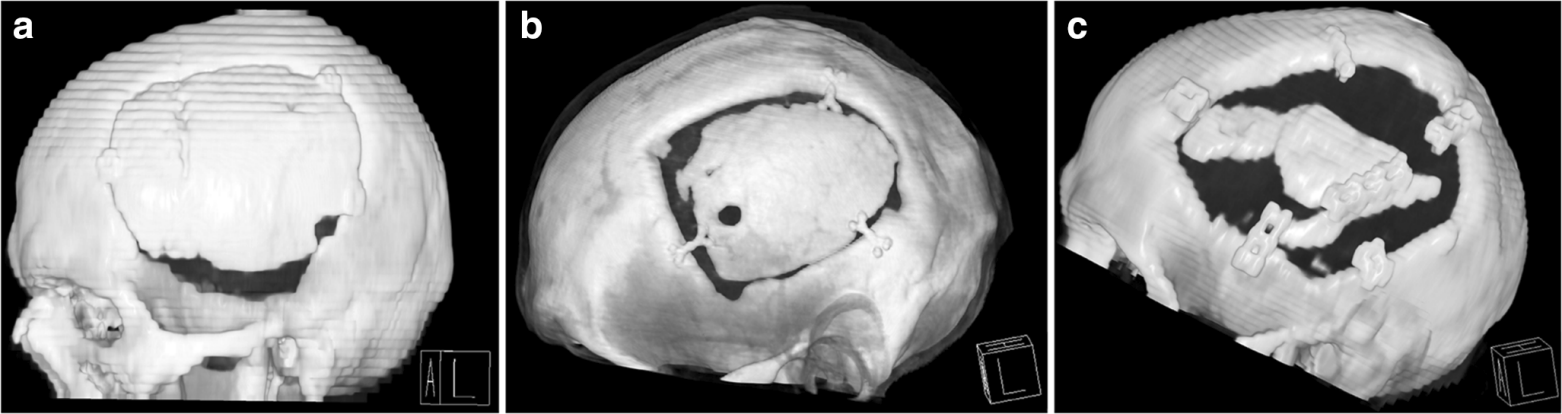
Three-dimensional cranial computed tomography reconstruction images showing examples of bone graft resorption graded as **a** mild: less than 50% of the graft circumference affected, **b** moderate: more than 50% of the graft circumference affected, and **c** severe: more than 50% of the original bone flap volume affected

The long-term outcome of secondary CP was assessed according to the occurrence of complications requiring revision surgery. Infections were diagnosed on the basis of the clinical appearance. Wounds with purulent secretion or signs of dehiscence were considered infected; positive bacterial cultures served as a confirmation. Cranial computed tomography (CCT) was used to diagnose postoperative intracranial bleeding. Among ACB implant patients, both conventional skull X-ray and CCT were used to identify signs of bone graft resorption. Resorption was defined as a progressive decrease of the postoperative bone flap volume, radiographically characterized by osteolytic bone gap widening, cortical irregularities, and perforations. The extent of resorption was evaluated based on a modified version of the classification proposed by Zhang et al. [12] and graded according to the distribution of osteolytic graft lesions as mild (less than 50% of the graft circumference affected), moderate (more than 50% of the graft circumference affected), or severe (more than 50% of the original bone flap volume affected). Examples illustrating the grades of bone graft resorption are shown in Fig. 1. Identification of a secondary dislocation of the implant usually relied on a physical examination and was confirmed with conventional skull radiographs or CCT. Indications for surgical revision procedures were determined clinically, either in cases where a conservative therapy was not possible (e.g., loss of the protective or cosmetic function of the implant) or in the presence of severe, life-threatening complications (e.g., infection or extensive intracranial hemorrhage). Emergency revisions were performed immediately, while the replacement of resorbed ACB implants was scheduled electively (occasionally up to 3 years later). Partial bone graft resorption and minor postoperative hematomas were managed conservatively.

Factors believed to influence the outcome of secondary CP included the implant material, gender, patient age, timing of CP, the severity of TBI as measured by the Glasgow Coma Scale (GCS) score at hospital admission, and the size of the cranial defect. To ensure a representative comparison of the cohorts, patients were stratified into clinically relevant groups. Patients under 18 and over 65 were considered at higher risk of complications and combined to achieve an adequate group size [5, 13]. The date of cranioplasty was used as an additional parameter to account for potential outcome differences related to changes in the clinical standards over time.

### Statistical analyses

Patient characteristics and variables were summarized in frequency tables. Continuous data were characterized using the median and the interquartile range (IQR). The primary endpoint was the time spent free from revision surgery (from the date of secondary CP to the date of revision surgery) and was estimated using Kaplan-Meier analysis. Univariate Cox regression analysis was used to quantify the impact of the implant material, patient age, gender, reconstruction interval, initial GCS score, size of the cranial defect, and the date of cranioplasty on the long-term outcome. Only variables with a *p* value of less than 0.1 were entered into a multivariate Cox regression model. Estimates of the effects were expressed as the hazard ratio (HR) and the 95% confidence interval (95% CI). The follow-up time was calculated from the date of the secondary CP to the last documented patient contact. Results were considered statistically significant if the *p* value was 0.05 or less. All statistical analyses were performed using IBM® SPSS® Statistics 23 for Mac.

## Results

### Study population and patient characteristics

**Fig. 2:**
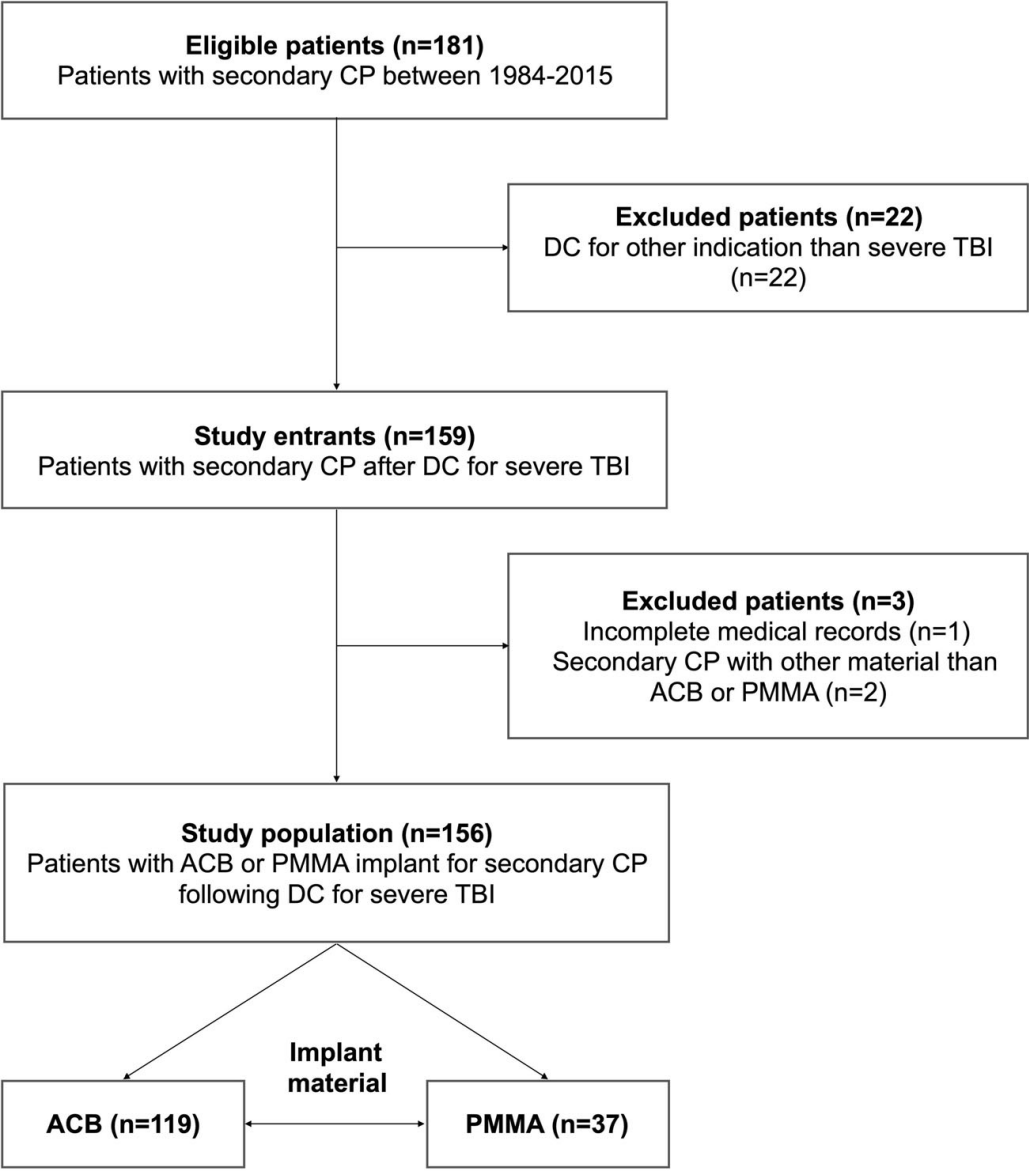
Flow chart illustrating the patient selection and cohort arrangement of patients who received secondary cranioplasty (CP) using autologous calvarial bone (ACB) or polymethylmethacrylate (PMMA) after decompressive craniectomy (DC) for severe traumatic brain injury (TBI) at the department of trauma surgery or neurosurgery between 1984 and 2015

Detailed information regarding patient selection and cohort arrangement is obtained from Fig. 2. A total of 181 patients received secondary CP between 1984 and 2015. DC was performed in 22 patients for indications other than severe TBI (e.g., stroke or aneurysmal subarachnoid hemorrhage). Following exclusions, the final study population consisted of 156 patients.

The median age at secondary CP was 41.8 years (26.1 to 55.2). One hundred and twenty-one (77.6%) patients were adults aged between 18 and 65 years; thirty-five (22.4%) patients were aged either below 18 (*n* = 17) or above 65 (*n* = 18). Of the 37 cranioplasties performed with PMMA a preoperatively fabricated, custom-made implant was only used in one patient (a 25-year-old woman) due to cosmetic issues, without observing any complications. Notably, patients operated between 1984 and 1999 received more frequently PMMA implants and had a longer median time interval between DC and cranial reconstruction than those operated between 2000 and 2015. In addition, PMMA was chosen more frequently in patients with smaller cranial defects than ACB, but less frequently in patients aged more than 65 years. Further descriptive statistics of the study population are shown in Table 1.

**Table 1:** Overview of patient characteristics stratified by the implant material used for secondary cranioplasty

Characteristic	All patients (*n* = 156)	ACB (*n* = 119)	PMMA (*n* = 37)
Age, years, median (IQR)	41.8 (26.1–55.2)	42.4 (28.1–55.4)	40.4 (21.8–54.9)
Age categories, years, *n* (%)			
18–65	121 (77.6)	91 (76.5)	30 (81.1)
< 18	17 (10.9)	12 (10.1)	5 (13.5)
> 65	18 (11.5)	16 (13.4)	2 (5.4)
Male gender, *n* (%)	129 (82.7)	98 (82.4)	31 (83.8)
Initial diagnosis^a^, *n* (%)			
SDH	107 (68.6)	85 (71.4)	22 (59.5)
EDH	45 (28.8)	32 (26.9)	13 (35.1)
ICH	45 (28.8)	35 (29.4)	10 (27.0)
SAH	22 (14.1)	18 (15.1)	4 (10.8)
Edema	10 (6.4)	8 (6.7)	2 (5.4)
Fracture	9 (5.8)	5 (4.2)	4 (10.8)
Initial GCS score, median (IQR)	5.5 (3.0–14.0)	5 (3.0–14.0)	7 (3.0–14.5)
GCS score categories, *n* (%)			
3–8	92 (59.0)	72 (60.5)	20 (54.1)
9–15	64 (41.0)	47 (39.5)	17 (45.9)
Cranial defect size^b^, cm^2^, median (IQR)	77 (60–99)	80 (63–108)	56 (30–72)
Defect size categories, cm^2^, *n* (%)			
< 80	69 (51.9)	48 (44.0)	21 (87.5)
≥ 80	64 (48.1)	61 (56.0)	3 (12.5)
Date of cranioplasty, *n* (%)			
1984–1999	23 (14.7)	9 (7.6)	14 (37.8)
2000–2015	133 (85.3)	110 (92.4)	23 (62.2)
Reconstruction interval, months, median (IQR)	5.8 (2.6–8.9)	4.6 (2.2–7.1)	9.5 (6.7–16.3)
1984–1999	9.0 (5.8–13.6)	5.8 (3.4–10.0)	12.9 (8.3–14.2)
2000–2015	5.3 (2.3–7.7)	4.4 (2.2–6.7)	8.9 (4.8–17.9)
Reconstruction interval categories, months, *n* (%)			
0–3	45 (28.8)	40 (33.6)	5 (13.5)
> 3	111 (71.2)	79 (66.4)	32 (86.5)
Complications, *n* (%)			
Resorption^c^	26 (16.7)	26 (21.8)	–
Mild	3 (1.9)	3 (2.5)	–
Moderate	8 (5.1)	8 (6.7)	–
Severe	15 (9.6)	15 (12.6)	–
Hematoma	7 (4.5)	5 (4.2)	2 (5.4)
Infection	5 (3.2)	5 (4.2)	0 (0.0)
Secondary dislocation	3 (1.9)	2 (1.7)	1 (2.7)
Revision surgery, *n* (%)	28 (17.9)	26 (21.8)	2 (5.4)
Follow-up, years, median (IQR)	0.9 (0.0–3.8)	0.9 (0.1–3.3)	0.4 (0.0–7.9)

*ACB* autologous calvarial bone, *EDH* epidural hematoma, *GCS* Glasgow Coma Scale, I*CH* intracerebral hemorrhage, *IQR* interquartile range, *PMMA* polymethylmethacrylate, *SAB* subarachnoid hemorrhage, *SDH* subdural hematoma

^a^More than one initial diagnosis is possible

^b^Data were only available for 133 patients

^c^Only occurs in autologous calvarial bone implants

### Complications

The frequencies of postoperative complications are shown in Table 1. A total number of 41 (26.3%) patients suffered at least one complication associated with secondary CP. All complications requiring surgical treatment occurred within 12 months after secondary CP, except for one patient who suffered secondary dislocation of the PMMA implant 3.7 years after initial surgery. Notably, the occurrence of surgical site infections and secondary dislocations always resulted in the need for a surgical revision procedure. A conservative management was possible in 2 of the 7 patients with postoperative hematomas and 11 of the 26 patients with bone graft resorption. Osteolytic ACB implants required re-operation in 3 of the 8 patients with moderate and 12 of the 15 patients with severe resorption.

### Long-term outcome predictors

**Fig. 3:**
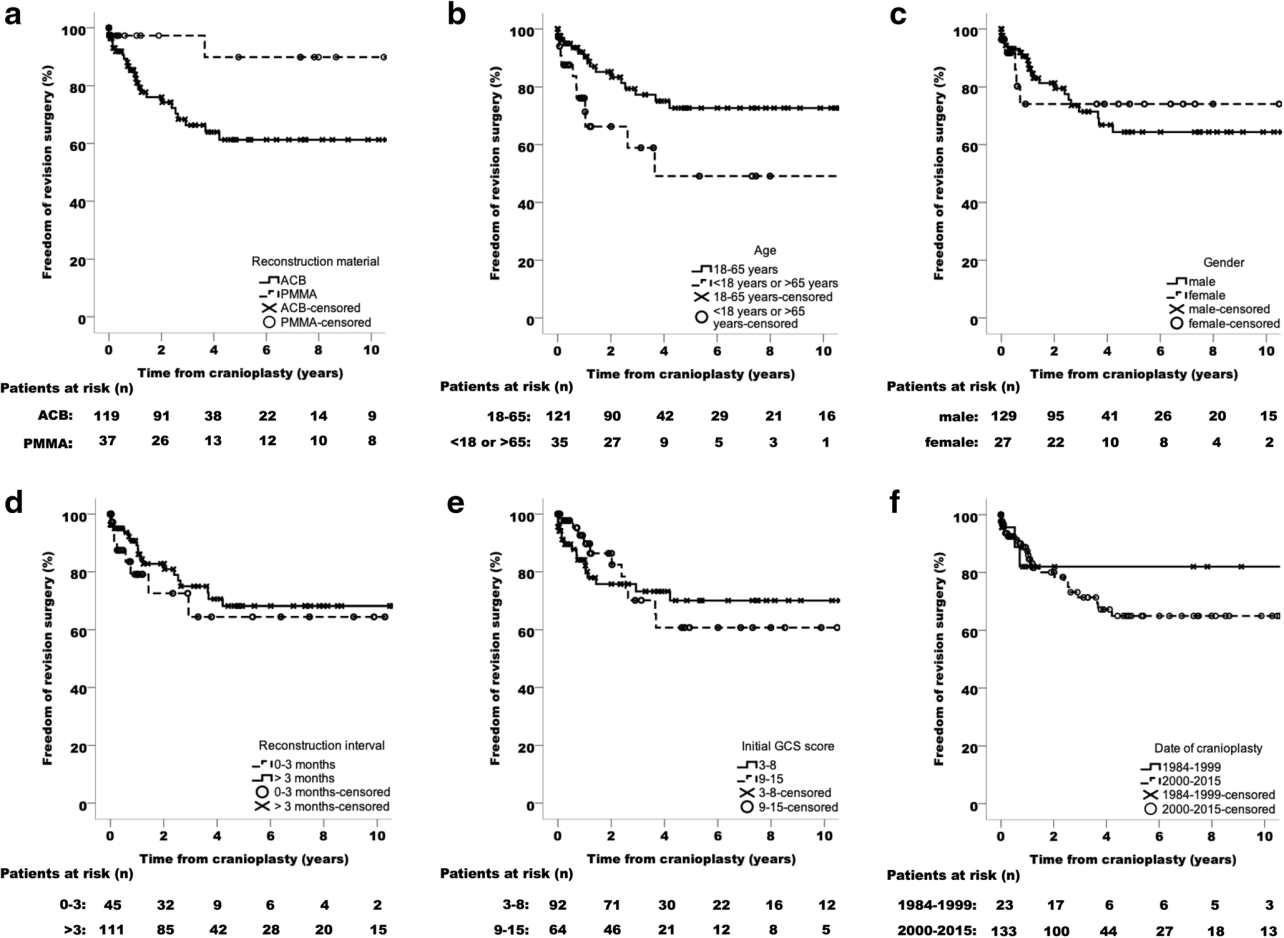
Kaplan-Meier curves estimating the time spent free from revision surgery at 10 years according to **a** the implant material, **b** the patient age, **c** the gender, **d** the reconstruction interval, **e** the initial Glasgow Coma Scale (GCS) score at hospital admission, and **f** the date of secondary cranioplasty

Univariate and multivariate Cox regression analyses of variables suggested to influence the outcome of secondary CP are shown in Table 2. A significant impact on the long-term outcome of secondary CP could be identified for the implant material and the patient age. Patients with PMMA implants had a lower risk to develop complications requiring surgical revision than those with ACB implants (HR 0.2, 95% CI 0.1 to 1.0, *p* = 0.04). The mean revision-free time interval was estimated to be 18.8 years (95% CI 16.2 to 21.4) for patients with PMMA implants and 9.1 years (7.6–10.7 years 95% CI) for patients with ACB implants. A similar effect could be observed for the patient age. Individuals aged between 18 and 65 years had a lower risk of requiring revision surgery after secondary CP than patients aged under 18 or over 65 years (HR 0.4, 95% CI 0.2 to 0.9, *p* = 0.02). For patients aged between 18 and 65, the mean event-free period was estimated to be 15.5 years (95% CI 13.3 to 17.7), whereas for those aged under 18 or over 65, it was estimated to be 9.1 years (95% CI 5.4 to 12.8). If patients aged under 18 and over 65 years are assigned to separate cohorts, they still had a higher risk of requiring revision surgery than patients aged between 18 and 65 years (under 18 years: HR 2.9, 95% CI 1.1 to 7.5, *p* = 0.03; over 65 years: HR 2.1, 95% CI 0.8 to 5.8, *p* = 0.14; Supplemental Tab. 1). No significant impact on the long-term outcome of secondary CP could be found for the gender (female versus male: HR 0.9, 95% CI 0.4 to 2.4, *p* = 0.88), reconstruction interval (> 3 months versus 0 to 3 months: HR 0.8, 95% CI 0.3 to 1.8, *p* = 0.53), the severity of TBI (GCS 9 to 15 versus 3 to 8: HR 0.9, 95% CI 0.4 to 2.0, *p* = 0.86), and the size of the cranial defect (≥ 80 cm^2^ versus < 80 cm^2^: HR 1.8, 95% CI 0.8 to 4.0, *p* = 0.16; Table 2 and Supplemental Tab. 1). Similarly, no outcome differences could be detected between patients who received their cranial implant before the year 2000 and those who were operated later (2000 to 2015 versus 1984 to 1999: HR 1.3, 95% CI 0.4 to 4.4, *p* = 0.63; Table 2 and Supplemental Tab. 1). Kaplan-Meier curves estimating the time spent free from revision surgery according to the investigated variables are shown in Fig. 3.

**Table 2:** Univariate and multivariate Cox proportional hazards regression models of variables suggested to influence the frequency of revision surgeries

Variable	Univariate Cox regression		Multivariate Cox regression^a^	
	HR (95% CI)	*p* value	HR (95% CI)	*p* value
Implant material				
ACB	1		1	
PMMA	0.3 (0.1–1.1)	0.06	0.2 (0.1–1.0)	0.04
Age, years				
< 18 or > 65	1		1	
18–65	0.4 (0.2–0.9)	0.03	0.4 (0.2–0.9)	0.02
Gender				
Male	1			
Female	0.9 (0.4–2.4)	0.88		
Reconstruction interval, months				
0–3	1			
> 3	0.8 (0.3–1.8)	0.53		
Initial GCS score				
3–8	1			
9–15	0.9 (0.4–2.0)	0.86		
Cranial defect size^b^, cm^2^				
< 80	1			
≥ 80	1.8 (0.8–4.0)	0.16		
Date of cranioplasty				
1984–1999	1			
2000–2015	1.3 (0.4–4.4)	0.63		

*ACB* autologous calvarial bone, *CI* confidence interval, *GCS* Glasgow Coma Scale, *HR* hazard ratio, *PMMA* polymethylmethacrylate

^a^Only variables with *p* < 0.1 in the univariate analysis were entered into the multivariate Cox regression model

^b^Data were only available for 133 patients

## Discussion

Our data demonstrates that both the implant material and the patient age at time of secondary CP are probably the most relevant parameters independently predicting the treatment success of secondary CP following DC for severe TBI. Patients who received PMMA implants had a significantly lower risk of undergoing revision procedures than patients treated with ACB implants. In contrast, pediatric (< 18 years) and geriatric (> 65 years) patients had an increased risk to suffer complications requiring surgical intervention. The adverse outcomes in this cohort were probably related to age-specific confounders. According to our data the gender, timing of secondary CP, severity of TBI and the size of the cranial defect had no influence on the frequency of revision surgeries. However, as the treatment regime was mainly adjusted to the patient’s clinical and neurological condition, subtle effects may not have been detected. Larger patient populations and longer follow-up periods would be required to investigate the impact of these variables more accurately.

### Implant material, patient age, and size of the cranial defect

ACB implants are frequently used as the first choice for secondary CP. Bhaskar et al. reported that Australian neurosurgical centers preferred to implant ACB in up to 96% of patients [14]. This is consistent with our cohort where ACB implants were used in 76% of cases. The preferential use of autologous bone is probably related to historic aspects and maintained by the fact that it is cheap, readily available, and considered to have an optimal biocompatibility [15]. However, current study results comparing the use of ACB implants and alloplastic materials for secondary CP are incongruent and there is no reliable data confirming the superiority of ACB implants. A systematic review of 13 studies recorded surgical site infections in 76 of 950 (8%) CPs performed with ACB and 61 of 632 (10%) procedures performed with alloplastic materials. The pooled OR was 0.81 for ACB implants but did not reach statistical significance (*p* = 0.57) [11]. For the interpretation of data, it is also important to consider that the preferential use of ACB for secondary CP might cause a selection bias disfavoring alloplastic materials [16]. In our study, PMMA implants were predominantly used in patients with limited availability of the ACB (e.g., caused by fragmentation or contamination due to open skull fractures). This could be associated with a more severe head trauma and a worse overall prognosis. Despite of this potential bias, our data indicates more favorable outcomes in patients with PMMA than ACB implants, if measured by the risk to develop complications requiring surgical revision procedures (HR 0.2, 95% CI 0.1 to 1.0, *p* = 0.04). The mean time without revision surgery was estimated at approximately 19 years in patients with PMMA implants, which was statistically 9.7 years longer than that of patients with ACB implants. Twenty-two percent of secondary CPs with ACB and 5% of cranial reconstructions with PMMA implants required surgical revision procedures in our study cohort. These revision rates are concordant with the data found in the literature, suggesting higher overall revision rates for ACB implants [16–19]. Thus, our data supports the evidence, which is increasingly questioning the safety of routinely used ACB for secondary CP.

However, providing a general recommendation towards the preferential use of alloplastic materials for secondary CP would be inadequate since the long-term outcome might be confounded by additional variables. According to our results, the estimated risk of undergoing a revision surgery was significantly lower in patients aged 18 to 65 than in the population of pediatric and geriatric patients (HR 0.4, 95% CI 0.2 to 0.9, *p* = 0.02). Several studies have already identified patient age to be a significant predictor of successful secondary CP [6, 8]. In the pediatric population, this could relate to higher rates of bone resorption [6]. Indeed, secondary CP in children is routinely performed using ACB. This might be argued by an at least 50% chance that the implant will become reintegrated, as reported by Grant et al. [5]. In contrast, alloplastic materials without osteoinductive properties will never become a “part of the body.” Due to the ongoing growth of the skull, pediatric patients are at a higher risk of requiring surgical revision procedures. Therefore, secondary CP using PMMA implants could not be generally recommended as the first-line treatment for cranial defects in children. The long-term outcome of secondary CP in the geriatric population is not well studied. However, systemic comorbidities are common and neurosurgical procedures are associated with a higher risk of postoperative complications and higher mortality rates compared to younger patients [13].

Finally, there is evidence of a higher incidence of bone graft resorption in patients with large cranial defects [20]. In our series, patients with skull defects of 80 cm^2^ or more also tended to have an increased risk to require surgical revision procedures (HR 1.8, 95% CI 0.8 to 4.0, *p* = 0.16). However, the preferential use of ACB implants in this subgroup of patients might have precluded statistical significance and contributed to worse outcomes in the ACB cohort.

### Gender

The influence of gender-specific parameters on the outcome of secondary CP is not well described. However, some authors have routinely compared outcome variables adjusted for the gender. No significant differences in rates of infections and overall complications could be found between male and female patients undergoing post-DC cranial repair [8, 9]. Consistent with the literature, our study could also not detect a significant influence of the patient gender on the frequency of surgical revision procedures (*p* = 0.88).

### Timing of secondary CP

The time interval between DC and secondary CP is a frequently investigated parameter. In children, it is recommended that the secondary CP is performed as early as possible due to the ongoing growth of the skull [21]. For the adult population study, results are heterogeneous and no generally accepted recommendation exists. Some evidence suggests that prolonging the interval is associated with increasing complication rates (9% < 3 months; 19% 3–6 months; 26% > 6 months); this statistically significant difference (*p* = 0.007) suggests a more favorable outcome for early secondary CP [8]. Contrasting evidence supports delayed secondary CP, with lower complication rates when it is performed more than 2 months after the DC (14% vs. 26%; *p* = 0.04) [10]. In our study, there was no correlation between the timing of the secondary CP and rates of revision surgeries (*p* = 0.53). However, the time interval was chosen according to the patient’s individual health condition, which might have biased our data and reduced the power to detect differences.

### Severity of TBI

The severity of TBI as a prognostic factor for the outcome of secondary CP has not yet been investigated extensively. There is limited evidence that the neurological outcome following severe TBI may influence the treatment success of secondary CP. Im et al. reported a significantly increased risk (HR 5.2, *p* = 0.04) of suffering surgical site infections after secondary CP in patients with poor neurological outcomes [22]. However, not all studies confirmed this association [23]. We stratified the severity of TBI according to the initial GCS score at hospital admission, which did not significantly correlate with the risk of revision surgery (*p* = 0.86). Indeed, the GCS score does not take other injuries potentially causing unconsciousness into account and therefore it probably cannot be considered the tool of choice to assess the severity of TBI [24]. A score assessing total injury severity would be a more sensitive parameter but was not available for all of our patients.

### Limitations

Due to the retrospective and non-randomized study, design data might be missing or biased and therefore study results have to be considered cautiously. This especially applies for the survival analyses, in which cases may have been missed or which might have overestimated the risk of suffering an event because patients experiencing severe complications probably had longer follow-up periods than those without. Moreover, important secondary outcome parameters including the neurological outcome and systemic comorbidities were not available for all patients. However, awareness of these limitations allowed for their consideration in the course of data interpretation. A larger study population and longer follow-up periods would have been necessary to detect small differences between the cohorts. We were able to identify variables with a strong impact on the outcome of secondary CP, but our study may have not been powered sufficiently to dissect subtle effects of some other important confounders. We are also aware that combination of patients aged under 18 and over 65 years into one cohort to achieve an adequate group size might have precluded the detection of outcome differences between both populations. For more accurate results regarding the long-term outcome of secondary CP large, prospectively designed clinical trials would be required, such as the recently published study by Honeybul et al. comparing titanium with ACB implants [25]. Finally, we used a modified classification to quantify bone graft resorption—adapted to the high prevalence of advanced cases in our cohort—limiting comparability to previous studies.

## Conclusion

The results of this study suggest that the implant material and the patient age are some of the most critical parameters independently determining the outcome of secondary CP. Considering the financial and personal health burdens associated with surgical revision procedures, secondary CP is required to be performed in the safest possible way. This will help to ensure an optimal long-term outcome and reduce postoperative complications to a minimum. Although our study had several inherent limitations, the use of PMMA was associated with lower revision rates than ACB, suggesting that alloplastic materials might provide a therapeutic benefit for the first-line treatment of selected patients with traumatic cranial defects.

## Electronic supplementary material

ESM 1 (DOCX 17 kb)
