# Supplementary material for: The impact of implant material and patient age on the long-term outcome of secondary cranioplasty following decompressive craniectomy for severe traumatic brain injury
Source: Acta Neurochir (Wien). 2020 Feb 5;162(4):745–53. doi: 10.1007/s00701-020-04243-7 (PMC7066309; doi:10.1007/s00701-020-04243-7)
Supplement: Supplementary file 1 — (DOCX 17 kb) [file 701_2020_4243_MOESM1_ESM.docx]

**Supplemental Tab. 1** Univariate and multivariate Cox proportional hazards regression models of variables suggested to influence the frequency of revision surgeries (with modified age groups)

| **Variable** |  |  | **Univariate Cox-regression** | | | |  | | **Multivariate Cox-regression^a^** | | |
| --- | --- | --- | --- | --- | --- | --- | --- | --- | --- | --- | --- |
|  |  |  | **HR (95% CI)** | | **p-value** | |  | | **HR (95% CI)** | | **p-value** |
| Implant material |  |  |  |  | |  | |  | |  | |
| ACB |  |  | 1 |  | |  | | 1 | |  | |
| PMMA |  |  | 0.3 (0.1-1.1) | 0.06 | |  | | 0.2 (0.1-1.0) | | 0.04 | |
| Age, years |  |  |  |  | |  | |  | |  | |
| 18-65 |  |  | 1 |  | |  | | 1 | |  | |
| <18 |  |  | 2.4 (0.9-6.1) | 0.07 | |  | | 2.9 (1.1-7.5) | | 0.03 | |
| >65 |  |  | 2.4 (0.9-6.5) | 0.09 | |  | | 2.1 (0.8-5.8) | | 0.14 | |
| Gender |  |  |  |  | |  | |  | |  | |
| male |  |  | 1 |  | |  | |  | |  | |
| female |  |  | 0.9 (0.4-2.4) | 0.88 | |  | |  | |  | |
| Reconstruction interval, months |  |  |  |  | |  | |  | |  | |
| 0-3 |  |  | 1 |  | |  | |  | |  | |
| >3 |  |  | 0.8 (0.3-1.8) | 0.53 | |  | |  | |  | |
| Initial GCS score |  |  |  |  | |  | |  | |  | |
| 3-8 |  |  | 1 |  | |  | |  | |  | |
| 9-15 |  |  | 0.9 (0.4-2.0) | 0.86 | |  | |  | |  | |
| Cranial defect size^b^, cm^2^ |  |  |  |  | |  | |  | |  | |
| <80 |  |  | 1 |  | |  | |  | |  | |
| ≥80 |  |  | 1.8 (0.8-4.0) | 0.16 | |  | |  | |  | |
| Date of cranioplasty |  |  |  |  | |  | |  | |  | |
| 1984-1999 |  |  | 1 |  | |  | |  | |  | |
| 2000-2015 |  |  | 1.3 (0.4-4.4) | 0.63 | |  | |  | |  | |

ACB, autologous calvarial bone; CI, confidence interval; GCS, Glasgow coma scale; HR, hazard ratio; PMMA, polymethylmethacrylate.

^a^Only variables with p<0.1 in the univariate analysis were entered into the multivariate Cox-regression model.

^b^Data were only available for 133 patients.
